# Seeing Is Believing: How Does the Surface of Silver Nanocubes Change during Their Growth in an Aqueous System

**DOI:** 10.1021/acs.nanolett.5c01276

**Published:** 2025-04-21

**Authors:** Qijia Huang, Dong Zhang, Hansong Yu, Yong Ding, Younan Xia

**Affiliations:** ‡ School of Chemistry and Biochemistry, Georgia Institute of Technology, Atlanta, Georgia 30332, United States; § The Wallace H. Coulter Department of Biomedical Engineering, Georgia Institute of Technology and Emory University, Atlanta, Georgia 30332, United States; ⊥ School of Materials Science and Engineering, Georgia Institute of Technology, Atlanta, Georgia 30332, United States

**Keywords:** silver nanocubes, seed-mediated growth, surface-enhanced Raman scattering, growth mechanism, surface reduction

## Abstract

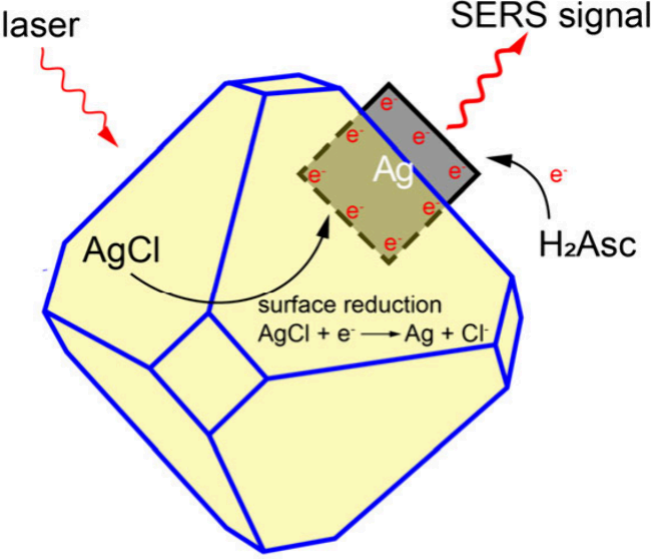

The seed-mediated growth involving cetyltrimethylammonium chloride (CTAC), silver trifluoroacetate (CF_3_COOAg), ascorbic acid (H_2_Asc), and Ag seeds covered by poly(vinylpyrrolidone) (PVP) in aqueous medium is a robust route to Ag nanocubes with tunable sizes. However, mechanistic details such as changes to the surface remain elusive. Herein, we address this issue by leveraging the high sensitivity and water compatibility of surface-enhanced Raman scattering (SERS). Our results reveal that the addition of CTAC results in ligand exchange between PVP and chloride and the further introduction of CF_3_COOAg leads to the deposition of AgCl on Ag seeds. The H_2_Asc subsequently introduced increases the electron density on the surface of the seeds due to electron transfer, as manifested by rapid and pronounced enhancement of the SERS signals from AgCl and CTA^+^. The electrons from H_2_Asc also enable reduction to directly transform AgCl in contact with Ag into Ag atoms and enlarge the seeds into nanocubes.

Silver (Ag) nanocubes have been extensively researched for their unique optical properties and applications in fields such as plasmonics,^1,2^ sensing,^3−5^ and catalysis.^6,7^ Of particular interest is their strong surface-enhanced Raman scattering (SERS) activity, which has been explored for sensitive detection of chemical species in close proximity to the surface.^8,9^ The optical properties of Ag nanocubes are strongly correlated with their size and the sharpness of their corners and edges. For instance, Ag nanocubes with an edge length of 80 nm were reported to give the strongest SERS activity when excited at a wavelength of 532 nm, with the optimal size increasing to 130 nm when switching to the excitation of 785 nm.^10^ Additionally, Ag nanocubes with sharper corners and edges are known to give stronger SERS signals relative to their truncated counterparts.^11^ For these reasons, protocols for producing size-controlled Ag nanocubes with sharp corners and edges have been actively explored.^12−18^ Among the methods reported in literature, polyol synthesis easily stands out as the most successful approach,^16,17^ but this method typically requires an elevated temperature around 150 °C, which tends to result in nanocubes with truncated corners and edges. In contrast, aqueous synthesis is better suited for generating and preserving sharp corners and edges on Ag nanocubes due to the use of a reaction temperature as low as 60 °C. To this end, seed-mediated growth in an aqueous medium has gained significant attention as a robust route to the synthesis of Ag nanocubes with sharp corners and edges, together with tunable edge lengths up to 110 nm.^12,15,19^

Despite the success in synthesis, the mechanistic details involved in the seed-mediated growth of Ag nanocubes in an aqueous medium remain elusive. For example, it is not clear if the precursor is reduced to atoms in the solution and then deposited onto the seeds, or if the precursor adsorbs on the surface of a seed, followed by reduction through autocatalysis.^20^ The latter mechanism is supposed to be more favorable due to the involvement of a lower energy barrier to the reduction. Additionally, very little is known about the changes to the surface of a seed during its evolution into larger nanocubes. Researchers have tried to address these issues by analyzing the reaction kinetics while characterizing the particles using techniques based upon electrochemistry, electron microscopy, and UV–vis spectroscopy.^21,22^ However, none of these techniques can directly detect and monitor the changes to the surface of Ag nanocubes during their growth to help elucidate the mechanistic details.

Vibrational spectroscopy techniques have been utilized to monitor the changes to the surface of colloidal metal nanoparticles induced by adsorption, desorption, aggregation, and/or chemical reactions. Specifically, Fourier transform infrared (FTIR) spectroscopy has been used to identify and track the chemical species adsorbed on the surface of Ag nanoparticles by analyzing their vibrational fingerprints.^23,24^ However, the applicability of FTIR to an aqueous system is limited due to the strong absorption of infrared radiation by water.^25^ Second-harmonic-generation (SHG) spectroscopy has also been adapted for real-time monitoring of seed-mediated growth of Ag and Au nanoparticles.^26,27^ In this case, the requirement for a high-intensity laser excitation in SHG may cause potential damage to the nanoparticles and even decompose the metal precursor, limiting the scope of application. In contrast, SERS offers a nondestructive alternative for the *in situ* characterization of chemical species in proximity to the surface of Ag or Au nanoparticles in an aqueous environment. In a recent study, we successfully adapted SERS to analyze the adsorbates on the surface of Ag nanocubes during a HCl-mediated polyol synthesis.^28^ Herein, we report the use of SERS to analyze the seed-mediated growth of Ag cubic seeds in an aqueous system. SERS data reveal the changes on the surface of seeds including ligand exchange, AgCl deposition, and electron transfer from reductant which led to a major enhancement in SERS signals. Collectively, our data points toward the dominance of autocatalytic reduction as the mechanism responsible for the seed-mediated growth of Ag nanocubes in an aqueous system.

Figure S1 shows a transmission electron microscopy (TEM) image of the Ag cubic seeds in the present work. They had an average edge length of 34.6 ± 6.3 nm, slightly larger than the seeds used in a prior study.^12^ We chose cubic seeds to ensure comparable SERS measurements throughout the growth. They were synthesized using the HCl-mediated polyol method and their surface was covered by a mixture of Cl^–^ ions and PVP.^29^ In a standard protocol for seed-mediated growth, aqueous cetyltrimethylammonium chloride (CTAC) was added into an aqueous suspension of the cubic seeds held at 60 °C, followed by the sequential introduction of silver trifluoroacetate (CF_3_COOAg) and ascorbic acid (H_2_Asc). The growth was then allowed to continue at 60 °C for 90 min. By adding different quantities of the seeds at a fixed amount of CF_3_COOAg, we were able to tune the size of the resultant nanocubes in a controllable fashion. Figure S2 shows TEM images of the Ag nanocubes synthesized using the standard protocol with the addition of 1000, 500, 300, and 100 μL of the same batch of seed suspension. The average edge lengths of the Ag nanocubes increased from 55.3 ± 2.7 to 60.5 ± 3.0, 79.2 ± 2.9, and 104.5 ± 5.8 nm, respectively. The strong correlation between the edge length of the nanocubes and the amount of the seeds confirms the direct participation of the seeds in the growth process, eliminating the involvement of solution reduction and homogeneous nucleation. As shown in Figure S3, the major localized surface plasmon resonance (LSPR) peaks of these nanocubes exhibited a red shift with increasing size. All spectra did not show the optical extinction peak associated with AgCl solid in the UV region, indicating its complete conversion to Ag by the end of a standard synthesis.

We then collected aliquots from the reaction mixture at different time points, rapidly cooling them to room temperature, and analyzed the samples by TEM, UV–vis, and SERS to gain insight into the growth process. Figure 1 shows the TEM images recorded from the samples obtained at *t* = 0 2, 10, 30, 60, and 90 min, respectively, of a standard synthesis conducted with the addition of 300 μL of the seed suspension. It is worth noting that the samples obtained at *t* = 0–10 min contained a large amount of AgCl nanoparticles. As documented in the literature,^30^ AgCl solid could be decomposed or reduced within seconds upon exposure to the electron beam of a transmission electron microscope. This explains the appearance of tentacle-like Ag nanocrystallites (indicated by square boxes) on the surface of some AgCl nanoparticles as shown in Figure 1, giving them an overall irregular morphology. Nevertheless, we could still resolve the truncated octahedral shape taken by the AgCl nanoparticles. There were a small number of the original Ag seeds (indicated by circular boxes) in the samples obtained at *t* = 0 and 2 min. As the synthesis progressed, the proportion of AgCl in the sample steadily decreased, while both the number and size of the Ag nanocubes increased. At *t* = 30 min, essentially all AgCl nanoparticles had disappeared. By *t* = 60 min, we obtained Ag nanocubes bearing sharp corners and edges, together with a uniform edge length of 81 nm. Figure S4 shows the UV–vis spectra recorded from aqueous suspensions of the samples obtained at different time points. For the sample obtained at *t* = 0 min, there was strong optical extinction in the range of 300–400 nm, which could be assigned to the AgCl nanoparticles. This extinction peak then decreased in intensity with time, and by *t* = 60 min, it essentially disappeared while the Ag nanocubes stopped growing into larger sizes. The major LSPR peak of the Ag nanocubes was shifted from 432 nm (the seeds) to 536 nm (*t* = 60 or 90 min) as their sizes were enlarged.

**Figure 1:**
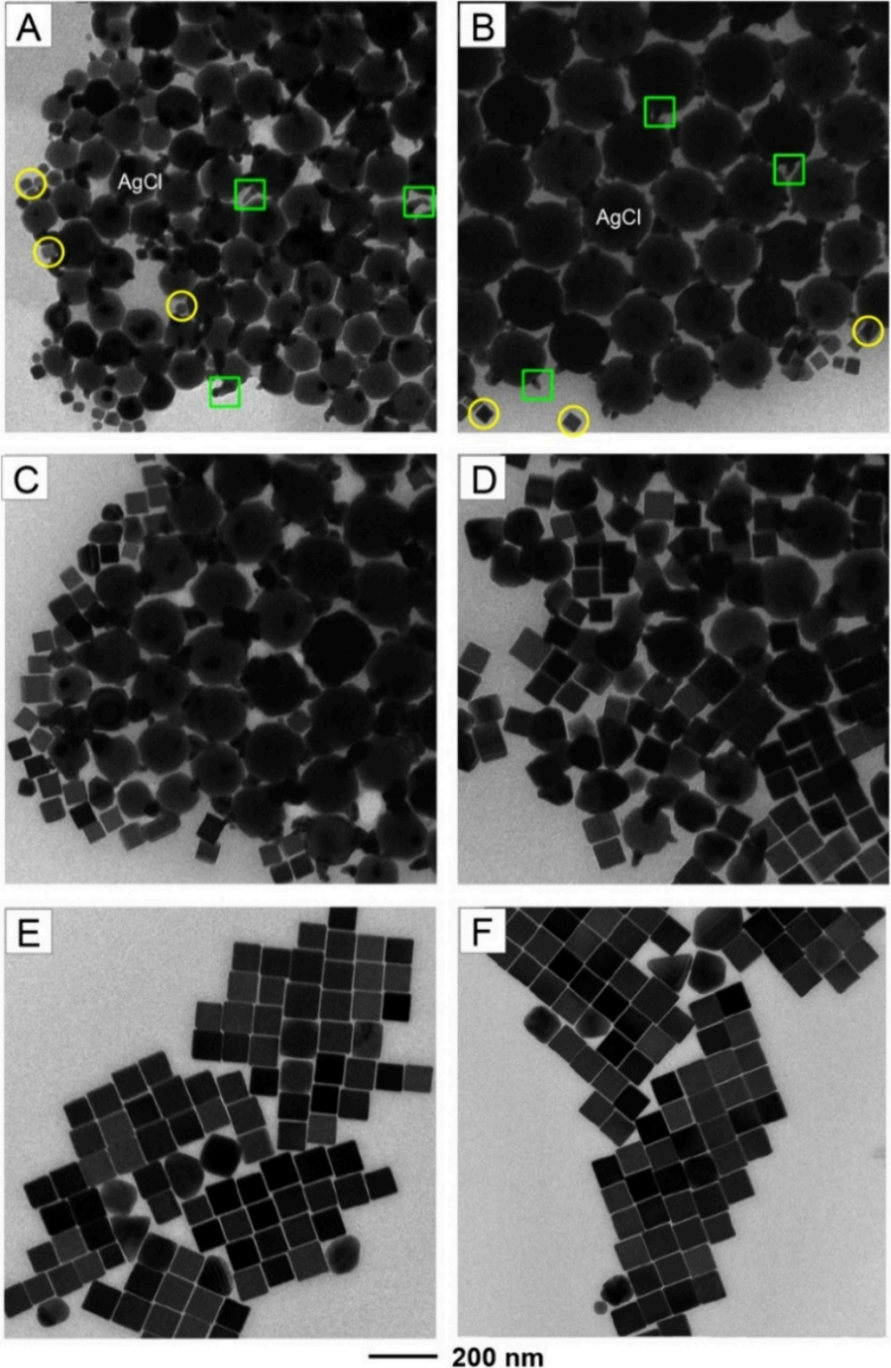
TEM images of the samples for SERS measurements obtained at different stages of a standard synthesis conducted in the presence of 300 μL of the seeds: (A) immediately after the introduction of CF_3_COOAg (*t* = 0 min) and (B) 2, (C) 10, (D) 30, (E) 60, and (F) 90 min after the introduction of H_2_Asc, respectively. In parts A and B, the Ag spikes formed on the surface of AgCl nanoparticles as a result of exposure to the electron beam are marked with square boxes, whereas the original Ag seeds are marked by circular boxes.

When the samples were analyzed by SERS, we observed distinct changes to the surface of the nanocubes. As shown in Figure 2, the Ag seeds exhibited the SERS spectrum typical of PVP-capped Ag nanocubes in an aqueous medium. The peak at 1761 cm^–1^ can be assigned to the C=O stretching mode of PVP (ν_C=O_), while the peaks at 927, 1403, and 2939 cm^–1^ are associated with the C–C ring breathing, CH_2_ scissoring, and CH_2_ stretching modes of PVP, respectively.^31^ We also observed a broad peak around 230 cm^–1^, which can be attributed to the stretching modes of Ag–Cl (ν_Ag–Cl_) and Ag–O (ν_Ag–O_).^28,32^ These two peaks are associated with the adsorption of Cl^–^ (during the polyol synthesis of the seeds) and the carbonyl group of PVP on the Ag surface, respectively. Upon the introduction of CTAC into the suspension, all SERS peaks associated with PVP disappeared, indicating the desorption of PVP from the surface of Ag seeds in the presence of CTAC at a high concentration. New peaks appeared at 760 and 1446 cm^–1^, and they could be assigned to the CH_3_ rocking (ρ_CH_3__) and N–CH_3_ bending (δ_N–CH_3__) modes of CTA^+^.^33^ Multiple peaks in the region of 2850–2975 cm^–1^ can be assigned to the C–H stretching modes (ν_C–H_) of CTA^+^. The spectral changes suggested that most of the PVP on the surface of the Ag seeds had been replaced by CTAC before CF_3_COOAg was added. Meanwhile, the peak at 230 cm^–1^ shifted to 240 cm^–1^ and became stronger in intensity, indicating increased Cl^–^ adsorption on the surface and desorption of the carbonyl group of PVP.

**Figure 2:**
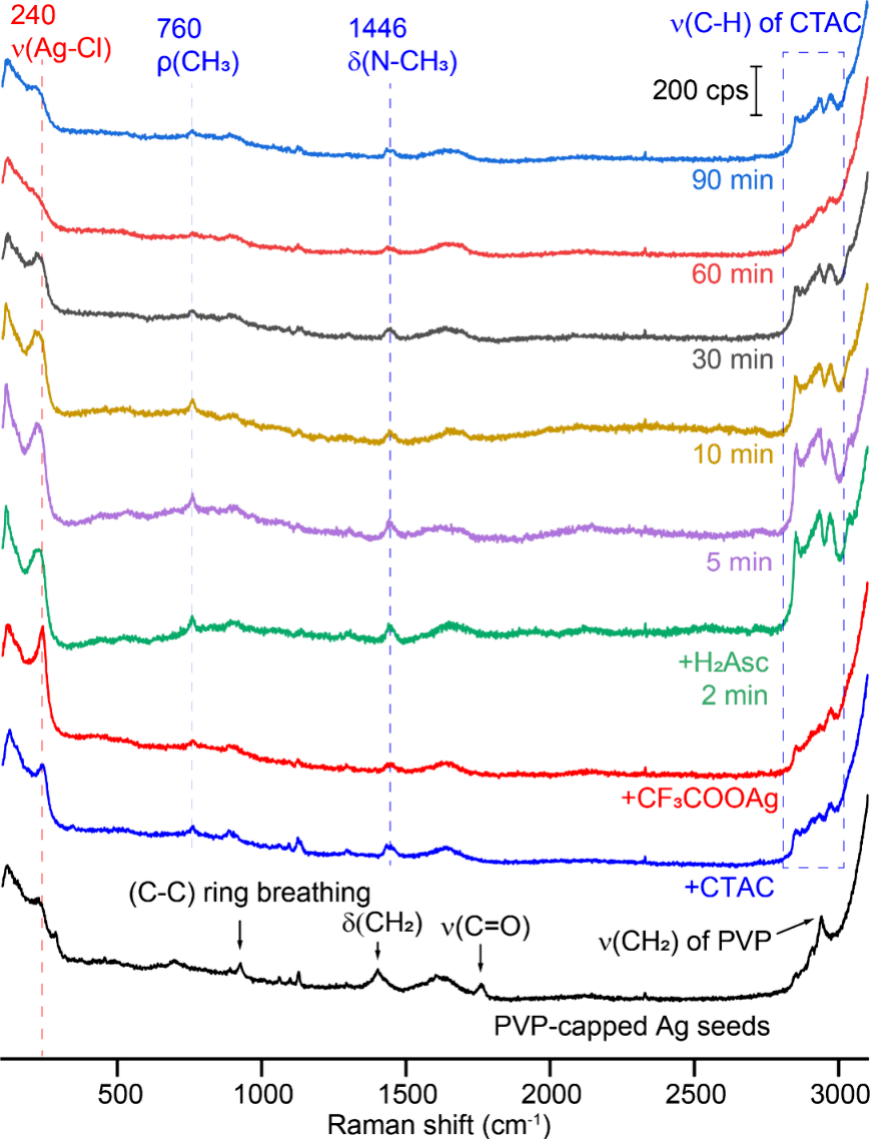
SERS spectra taken from the samples obtained at different stages of a standard synthesis conducted with the addition of 300 μL of the seed suspension.

Upon the introduction of CF_3_COOAg, the intensity of ν_Ag–Cl_ increased noticeably. The corresponding TEM images in Figure 1 indicated the formation of a large amount of AgCl nanoparticles. In addition to the enhancement in SERS signals caused by the presence of more AgCl on the surface of the Ag seeds, we argue that the ordinary Raman signals from the AgCl solid itself also contributed to the augmentation of peak intensity. It is worth mentioning that the SERS signals of CTA^+^ slightly decreased after the introduction of CF_3_COOAg. This trend indicates that less CTA^+^ were attached to the surface of the Ag seeds as most of them were covered by AgCl solid. It also ruled out the potential photoreduction of AgCl under laser irradiation during the SERS measurement. Otherwise, we would expect to observe stronger SERS signals of CTA^+^ than those observed for the sample before adding CF_3_COOAg. At *t* = 2 min after the introduction of H_2_Asc, we observed appreciable enhancement to the SERS signals from both AgCl and CTA^+^, and the AgCl peak also shifted from 240 to 234 cm^–1^. This augmentation indicates the transfer of electrons from H_2_Asc to Ag surface for the activation of chemical enhancement. A comparison of the spectra recorded from *t* = 2–90 min suggests that the intensities of SERS signals from all surface species gradually decreased as the reaction proceeded.

According to the literature, the SERS activity of Ag nanocubes under 532 nm excitation should gradually increase with particle size, reaching maximum at an edge length around 80 nm.^10^ However, compared to the SERS spectrum of Ag seeds (ca. 36 nm), the peak areas of ν_Ag–Cl_ and N–CH_3_ bending in the spectra at *t* = 60 and 90 min (ca. 81 nm) did not show any significant increase. This discrepancy could be attributed to the involvement of hot spots for SERS and the preferred adsorption of CTAC on the side faces of Ag nanocubes. Calculations based on the finite-element method indicated that the hot spots of a slightly truncated Ag nanocube were located on the corners and edges rather than the side faces.^34^ As such, the CTAC molecules adsorbed on the side faces would benefit less from the strong enhancement typically associated with the corners and edges, explaining why there was no major enhancement in SERS signals as the nanocubes were increasingly enlarged. We also conducted a control experiment by voiding Ag seeds from the standard protocol. As shown in Figure S5, the SERS signals from CTA^+^ and AgCl, as well as their magnitudes of enhancement, were significantly weaker even after the growth had been initiated compared to the SERS spectra in Figure 2. These observations confirmed the significant enhancement in SERS signals after adding H_2_Asc mainly came from the preformed Ag seeds rather than Ag nanocrystallites formed *in situ* on the AgCl. Further details are provided in the Supporting Information.

We investigated the growth of the same batch of Ag seeds using UV–vis spectroscopy at room temperature to confirm that no significant growth occurred during sample preparation and SERS measurement. As shown in Figure S6, the major LSPR peak shifted from 432 to 465 nm after 4 h into the synthesis indicating slight enlargement of the seeds. It was much slower relative to the case of standard synthesis conducted at 60 °C, where the peak reached a similar position of 465 nm within just 2 min. We also argued that the AgCl nanoparticles could not be photoreduced during the SERS measurement, which was conducted at room temperature under the excitation of a 532 nm laser. According to the literature, the photoreduction of silver halides can be attributed to Frenkel defects, which enable the reduction of Ag^+^ ions to Ag atoms when photoelectrons, generated under the excitation of photons with an energy exceeding the band gap, migrate to the trap states.^35,36^ The excitation light we used had a lower photon energy (532 nm, about 2.33 eV) than the energy (about 3 eV) required to initiate the photoreduction of AgCl solid, which has a band gap of 3.28 eV.^37^ Additionally, photocurrent measurements indicated that there was only a minimal reduction of Ag/AgCl material under 532 nm irradiation, as evidenced by the nearly absent cathodic photocurrents.^38^ Moreover, due to the presence of preformed Ag seeds in our system, most of the photoelectrons should be accepted by the Ag seeds embedded in the AgCl solid rather than being used to directly reduce AgCl solid for the generation of new Ag nuclei. This phenomenon resembles the concentration principle in photography by which a Ag nucleus will take all of the photoelectrons once it is formed in/on the AgCl or AgBr grain, preventing the formation of additional Ag nuclei on the same grain.^39^ As a result, the impact of laser exposure on the sample during SERS measurement should be negligible.

Based on the results from TEM, UV–vis, and SERS studies, we came up with a mechanism to account for the growth of Ag nanocubes in an aqueous system. Figure 3 shows a schematic of the mechanistic details. Specifically, the PVP on the surface of the seeds is exchanged with the Cl^–^ ions from CTAC used in excess, followed by the formation of AgCl octahedral nanoparticles upon the introduction of CF_3_COOAg. At this point, the resolved AgCl peak comes from both the SERS signals of the Cl^–^ adsorbed on the surface of the seeds and the Raman signals from the AgCl solid. Since heterogeneous nucleation has a lower energy barrier than that of homogeneous nucleation, the AgCl nanoparticles are supposed to nucleate and grow from the existing Ag cubic seeds. As a result, for the samples collected in the early stage of a synthesis, the Ag seeds are expected to exist in three different states: partially covered by AgCl (Ag-AgCl); fully encapsulated in AgCl (Ag@AgCl), and not covered by AgCl (naked Ag seeds). After H_2_Asc was added, the SERS signals from both CTA^+^ and AgCl increase significantly due to the chemical enhancement arising from the increase in electron density on the surface of the Ag seeds. For the seed partially embedded in AgCl solid, they can directly accept electrons from H_2_Asc and quickly reduce the Ag^+^ ions in the AgCl solid around the seed. The surface of the Ag seed becomes more electron-rich, with an electron density greater than the state before the addition of H_2_Asc, causing significant enhancement to the SERS signals. Similar to the development of a photographic film, it is the Ag-AgCl nanoparticles that will be immediately activated for growth upon the introduction of H_2_Asc. Meanwhile, as the reaction progressed, the AgCl was gradually consumed through reduction and dissolution, leading to a transformation of Ag@AgCl into Ag-AgCl. As shown in Figures 1 and S4, all of the AgCl on the Ag-AgCl nanoparticles has been reduced and converted to Ag by *t* = 30 min for the formation of enlarged nanocubes. Afterward, the growth will be continued through Ostwald ripening, by which the Ag^+^ ions and Ag atoms contained in the Ag@AgCl nanoparticles and the Ag atoms in the naked seeds are continuously transferred to the growing nanocubes.

**Figure 3:**
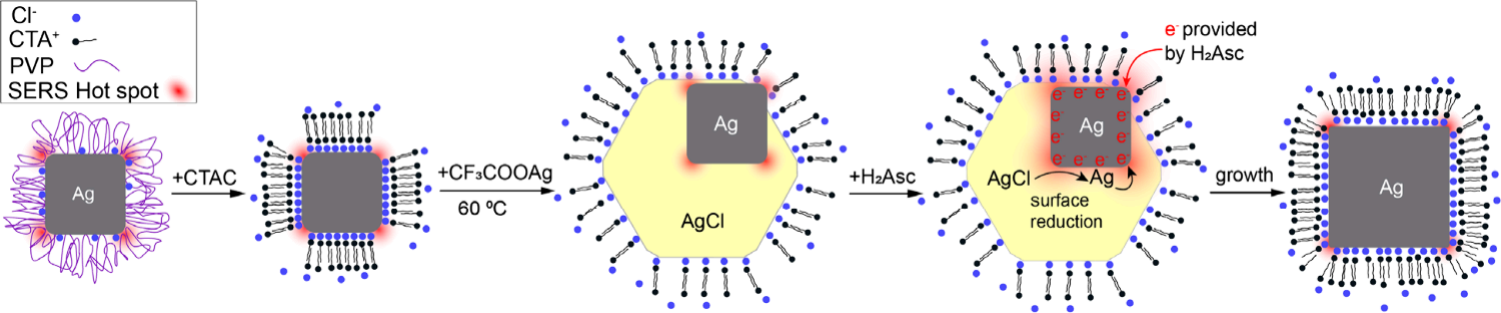
Schematic illustration showing the surface and structural changes involved in the seed-mediated growth of Ag nanocubes in an aqueous medium sequentially supplied with CTAC, CF_3_COOAg, and H_2_Asc.

To gain a more accurate understanding of the surface of Ag nanocubes during their growth, we directly recorded SERS spectra from the reaction mixture *in situ* at a temperature close to 60 °C using the setup shown in Figure S7. The concentrations of all chemical species were kept the same as those used in the *ex situ* SERS measurements. As shown in Figure 4A, the introduction of H_2_Asc solution led to a rapid increase in SERS signals within 40 s, agreeing with the result observed at *t* = 2 min during the *ex situ* measurement. Figure 4B shows that the SERS signals of both AgCl and CTA^+^ increased by approximately 4 times within 1 min. Since the signal enhancement occurred within a very short period of time after the growth was initiated, the Ag nanocrystallites newly formed on the surface of AgCl might not be large enough to provide hot spots based on the discussion regarding Figure S5. The fine temporal resolution of the *in situ* measurement provides additional evidence to confirm the rapid formation of structural features responsible for signal enhancement, in addition to the slight attenuation of SERS signals over the first 10 min observed during the *ex situ* measurement.

**Figure 4:**
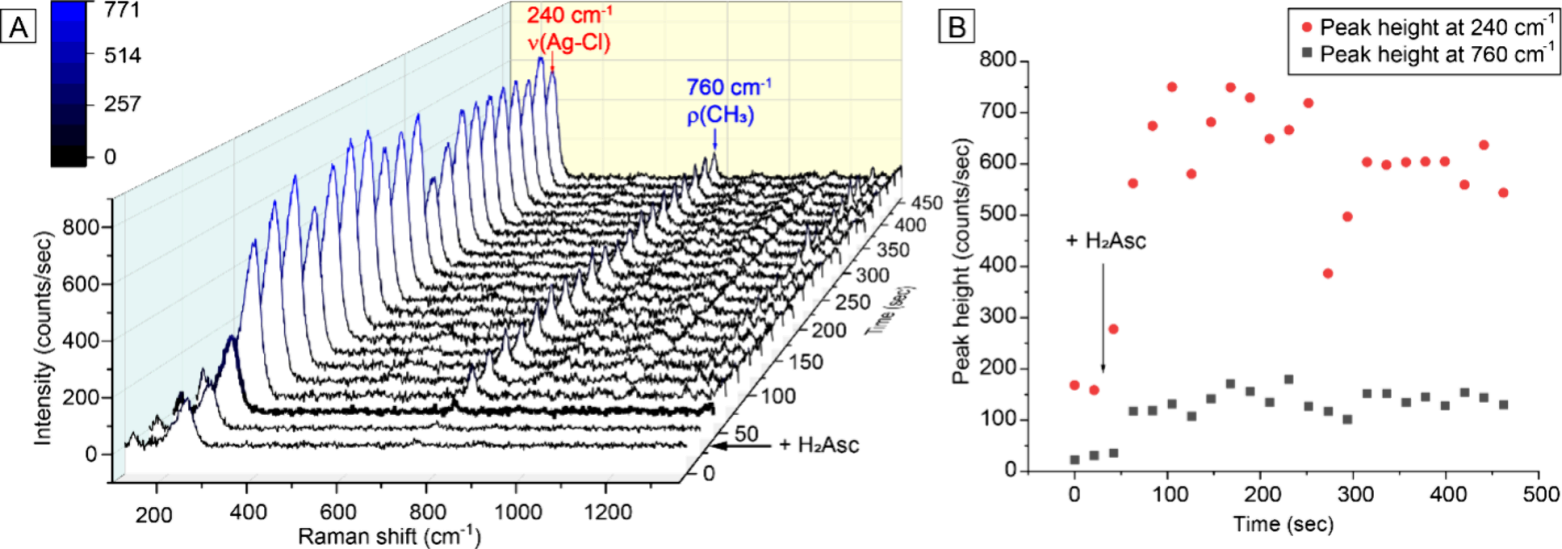
(A) *In situ* SERS spectra taken from the reaction mixture at different stages (*t* = 0–450 s) after the introduction of H_2_Asc for a standard synthesis conducted with 60 μL of the seeds. (B) Plots of the heights for the ν_Ag–Cl_ peak at 240 cm^–1^ and the CH_3_ rocking peak at 760 cm^–1^, from the N^+^(CH_3_)_3_ group of CTAC, derived from the *in situ* SERS spectra shown in the left panel.

Since the mixing between H_2_Asc and the reaction solution was somewhat retarded relative to the standard synthesis that involved magnetic stirring, the reaction was expected to progress at a slower rate during the *in situ* measurement. Additionally, the enhancement was much greater than the SERS signal enhancement expected from increased sharpness of the nanocubes.^11^ Therefore, the rapid appearance of enhanced SERS signals should be better explained by the increase in surface electron density, which further supports surface reduction as the primary mechanism for the seed-mediated growth. The gradual decrease in the SERS signal over the first 10 min could be attributed to the corresponding decline in the reducing agent concentration as the reaction proceeded. It is worth noting that the abnormal signal attenuation observed during the *in situ* measurement can be attributed to the occasional formation of bubbles during the injection of H_2_Asc solution. Altogether, the data from both *in situ* and *ex situ* measurements are in good agreement, offering a solid foundation for understanding the mechanistic details involved in seed-mediated growth of Ag nanocubes.

In summary, we have demonstrated the use of SERS as a sensitive technique to probe the surface chemistry of Ag nanocrystals during the synthesis of Ag nanocubes with enlarged sizes up to 100 nm through seed-mediated growth in an aqueous system. Our results from both *in situ* and *ex situ* measurements reveal the removal of PVP from the surface of the seeds upon the introduction of CTAC, followed by the formation of AgCl solid upon the addition of CF_3_COOAg. Major SERS enhancement was observed upon the introduction of H_2_Asc, which can be primarily attributed to the increase in electron density on the surface of the Ag seeds due to the acceptance of electrons from H_2_Asc. Collectively, this work offers invaluable insights into the compositional changes on the surface of Ag nanocubes during their growth in an aqueous phase. It also provides a potential route augmenting the SERS signal by increasing the electron density on the surface of Ag nanocrystals through controlled chemical processes.

## Supporting Information Available

Experimental section, TEM images and UV–vis spectra of Ag seeds and Ag nanocubes synthesized with different amounts of seeds, UV–vis spectra of the samples for SERS measurements, Raman/SERS and UV–vis spectra showing the reduction of AgCl in the absence of preformed Ag seeds, and photographs and infrared image of the setup used for *in situ* SERS measurement (PDF)
