# Supplementary material for: Seeing Is Believing: How Does the Surface of Silver Nanocubes Change during Their Growth in an Aqueous System
Source: Nano Lett. 2025 Apr 21;25(17):7115–20. doi: 10.1021/acs.nanolett.5c01276 (PMC12046588; doi:10.1021/acs.nanolett.5c01276)
Supplement: Supplementary file 1 — nl5c01276_si_001.pdf [file nl5c01276_si_001.pdf]

Supporting Information

Seeing Is Believing: How Does the Surface of Silver Nanocubes Change during Their Growth in an Aqueous System

Qijia Huang,[a†] Dong Zhang,[b†] Hansong Yu,[c] Yong Ding,[c] and Younan Xia[ab*]

[a]School of Chemistry and Biochemistry, Georgia Institute of Technology, Atlanta, Georgia 30332, United States

[b]The Wallace H. Coulter Department of Biomedical Engineering Georgia Institute of Technology and Emory University, Atlanta, Georgia 30332, United States

[c]School of Materials Science and Engineering, Georgia Institute of Technology, Atlanta, Georgia 30332, United States

[†]These authors contributed equally to this work.

*Corresponding authors. E-mails: younan.xia@bme.gatech.edu

Experimental Section

Chemicals and materials. Ethylene glycol (EG, >99 %, Lot No. 0000160068) was ordered from J. T. Baker. Cetyltrimethylammonium chloride solution (CTAC, 25 wt% in H_2O, Lot No. STBH7200), silver trifluoroacetate (CF_3COOAg, >99.99%, Lot No. MKBZ0931V), ascorbic acid (H_2Asc, 99.7 %, Lot No. SLBS0713V), acetone (>99.5%), sodium hydrosulfide hydrate ($NaSH \cdot xH_2O$, Lot No. SHBP0761), and poly(vinyl pyrrolidone) (PVP, $M_w \approx 55{,}000$, Lot No. MKCD1968) were all purchased from Sigma-Aldrich. Hydrochloric acid (trace metal grade, Lot No. 24006167) was obtained from Fisher Chemical. All the chemicals were used as received. Deionized (DI) water dispersed from a Millipore water purification system (with a resistivity of 18.2 MΩ·cm at room temperature) was used in all experiments.

Synthesis of Ag nanocubes to be used as the seeds. We followed a recently refined polyol method.[31] In a typical synthesis, 20 mL of EG was added into a 250-mL three-neck round-bottom flask capped with ground glass stoppers and preheated under magnetic stirring (260-320 rpm) in an oil bath to a pre-set temperature of 150 ℃. The solution was held at this temperature for about 20 min, followed by the quick injection of 0.24 mL of NaSH solution in EG (3 mM). After 2 min, 2.0 mL of HCl solution in EG (3 mM) was introduced, followed by the addition of 5.0 mL of PVP solution in EG (20 mg/mL). After another 5 min, 1.6 mL of CF_3COOAg solution in EG (282 mM) was added in one shot and we started to monitor the main LSPR peak position of the reaction mixture using a UV-vis spectrometer. Specifically, a few drops of the reaction solution were withdrawn from the flask using a glass pipet and diluted with water in a cuvette, followed by the collection of its extinction spectrum. When the main LSPR peak reached 430 nm, the reaction was immediately quenched by immersing the flask in an ice bath. The solid products were crushed out with acetone ($V_{sample}/V_{acetone}$=1:3) and then collected by centrifugation, followed by washing with water three times to help remove the excess PVP. Finally, the nanocubes were dispersed in water at a concentration of 1.2×10^{12} for seed-mediated growth.

Seed-mediated synthesis of Ag nanocubes with enlarged sizes. In a standard protocol, 0.02 g of the CTAC solution and 4.4 mL of water were mixed in a 20-mL vial. Next, different amounts (1000, 500, 300, and100 μL) of the seed suspension was added, followed by 100 μL of 0.1 M aqueous CF_3COOAg. The vial was capped and immersed in a water bath held at 60 °C. After 20 min, 1 mL of 0.1 M aqueous H_2Asc was added, and the capped mixture was magnetically stirred for another 90 min. The reaction was allowed to proceed for 90 min and the solid products were

collected by centrifugation at 7000 rpm for 10 min. After carefully removing the supernatant, the Ag nanocubes were re-suspended in 5 mL of 1 mM aqueous CTAC for further use. During the growth, aliquots were also sampled from the reaction mixture at different time points from 2–90 min to check the LSPR peak positions of the nanocubes using a UV-vis spectrometer.

Raman/SERS measurement during seed-mediated growth of Ag nanocubes. In a standard protocol, 300 μL of the seed suspension and 4.4 mL of water were mixed in a 20-mL vial. Next, 0.02 g of the aqueous CTAC and 100 μL of 0.1 M CF_3COOAg solution were introduced sequentially. The capped vial was kept in a water bath at 60 °C for 20 min. After withdrawing 200 μL from the mixture for Raman measurement, 1 mL of 0.1 M aqueous H_2Asc was added, and the solution was magnetically stirred in the capped vial for up to 90 min. Aliquots of 200 μL were withdrawn from the reaction mixture at different time points and transferred to a 1.5-mL tube immersed in an ice bath to quench the reaction. Then, 25 μL of each quenched reaction solution was transferred into a cell made of poly(dimethyl siloxane) (PDMS), covered with a glass coverslip, and placed on the sample stage of the microscope. We collected Raman/SERS spectra in the extended mode at an excitation wavelength of 532 nm, together with a 100× objective lens, a laser power at 25 mW, and a collection time of 10 s.

SERS measurement of the reaction solution in the absence of Ag seeds. In a typical study, we followed the standard protocol for Raman/SERS measurement, except that the suspension of Ag seeds was replaced with pure water. We collected SERS spectra in the extended mode at an excitation wavelength of 532 nm, together with a 100× objective lens, a laser power at 25 mW, and a collection time of 10 s.

In situ SERS monitoring of the seed-mediated growth process. In a typical study, 0.004 g of the CTAC solution and 0.88 mL of water were mixed in a 20-mL vial. Next, 60 μL of the seed suspension and 20 μL of 0.1 M aqueous CF_3COOAg were introduced sequentially. The capped vial was kept in a water bath at 60 °C for 20 min. Some of the mixture was then transferred into a PDMS cell covered with a glass coverslip, placed on a petri dish filled with hot (60 °C) water, and placed on the sample stage of the microscope. During *in situ* SERS measurement, 0.2 mL of 0.1 M aqueous H_2Asc was injected into the cell, and the reaction solution was mixed by drawing and injecting the liquid three times with a pipette. The temperature of the setup was maintained by heat exchange with the water in the petri dish. We collected Raman/SERS spectra with laser excitation at 532 nm and 50 mW power, together with a 100× objective lens. Each spectrum was recorded in

the static mode, accumulating 5 scans from 100 to 1360 cm^{-1} with an exposure time of 1 sec per scan. Spectra were collected every 20 s, and the laser was blocked between collections to minimize exposure of the sample.

Characterizations. The TEM images were captured using a microscope (HT7700, Hitachi) operated at 120 kV. The UV−vis spectra were recorded on a spectrophotometer (Cary 60, Agilent). Metal contents were measured using an inductively-coupled plasma mass spectrometer (ICP-MS, NexIon, PerkinElmer). The Raman and SERS spectra were recorded using a Renishaw inVia Raman Spectrometer (Wotton-under-Edge, U.K.) integrated with a Leica microscope (Wetzlar, Germany).

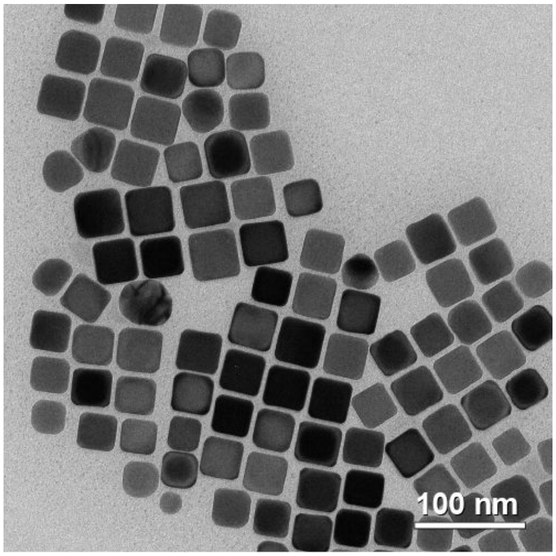

Figure S1. TEM image of the 36-nm Ag nanocubes serving as seeds for the growth.

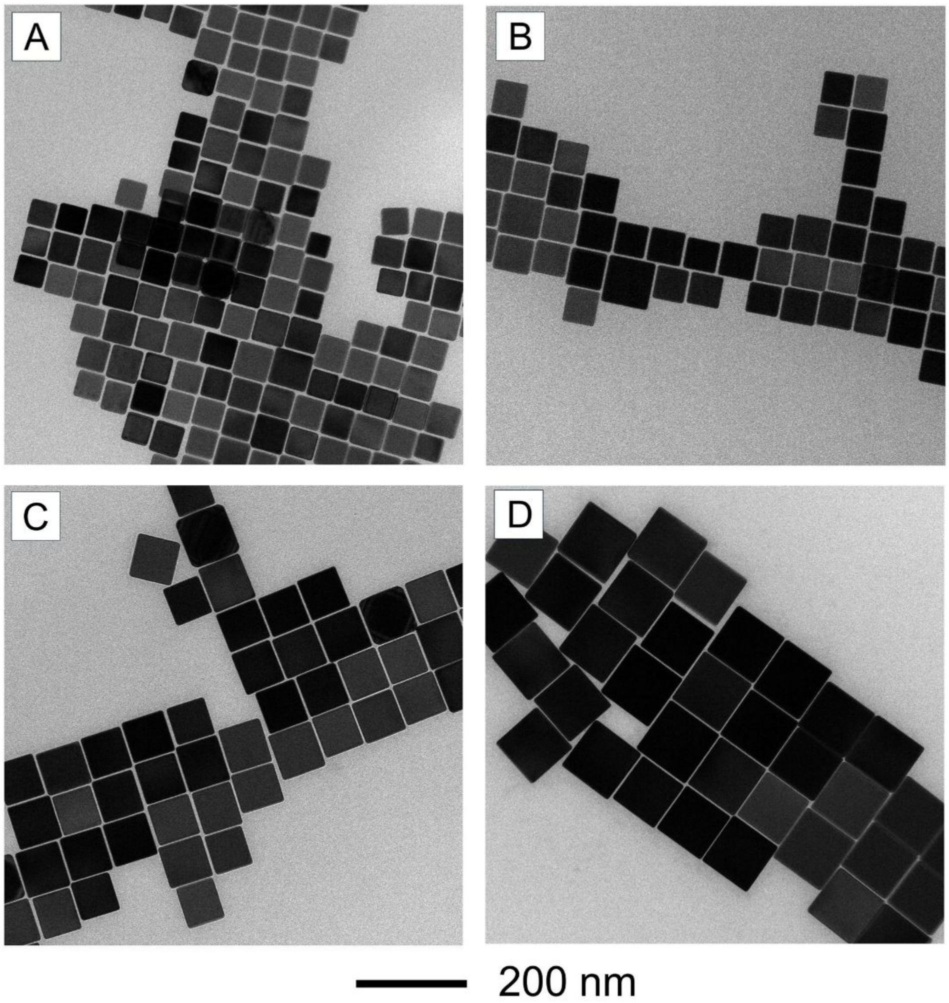

Figure S2. TEM images of the Ag nanocubes synthesized using the standard protocol for seed-mediated growth with (A) 1000, (B) 500, (C) 300, and (D) 100 μL of the seed suspension. The average edge lengths of the Ag nanocubes increased from 55.3±2.7 to 60.5±3.0, 79.2± 2.9, and 104.5±5.8 nm, respectively, as the amount of the seed suspension was reduced.

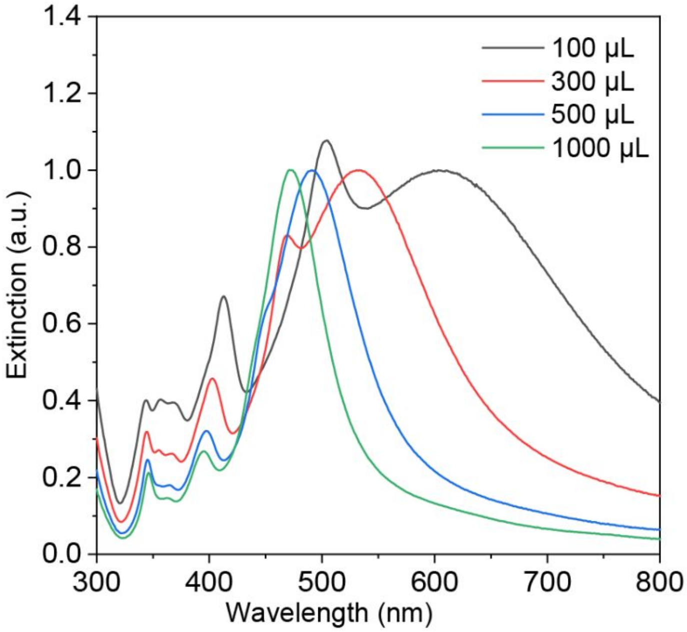

Figure S3. UV-vis extinction spectra recorded from aqueous suspensions of Ag nanocubes with enlarged sizes, which were synthesized using the standard protocol by adding 1000, 500, 300, and 100 μL of the seed suspension. The major localized surface plasmon resonance (LSPR) peaks of these nanocubes were located at 474, 492, 532, and 605 nm, respectivelyThe products obtained from the synthesis with the addition of 300 or 100 μL of the seed suspension showed a shoulder peak next to the major peak and this shoulder peak could be attributed to the quadrupole charge distribution

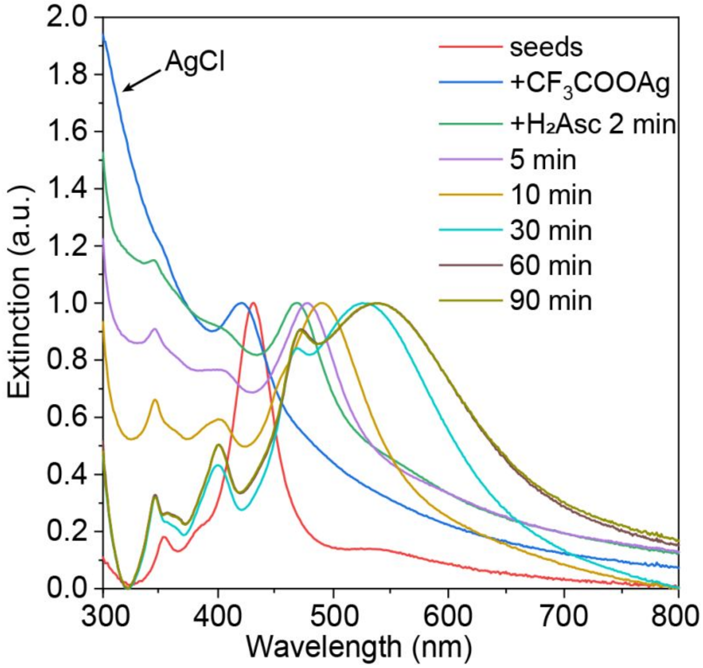

Figure S4. UV-vis spectra of the samples for SERS measurements obtained at different stages of a standard synthesis conducted with the use of 300 μL of the seed suspension. The broad shoulder peak observed in the range of 500−600 nm for the seeds could be attributed to the introduction of CTAC. The presence of CTAC at a high concentration could lead to surface exchange between PVP and Cl^- ions, as well as compression of the electric double layer. As a result, the electrostatic repulsion among the particles and the stearic hindrance caused by surface-bound PVP would be compromised, inducing particle aggregation. However, the aggregation seemed to be reversible as confirmed by the good uniformity of the final nanocubes.

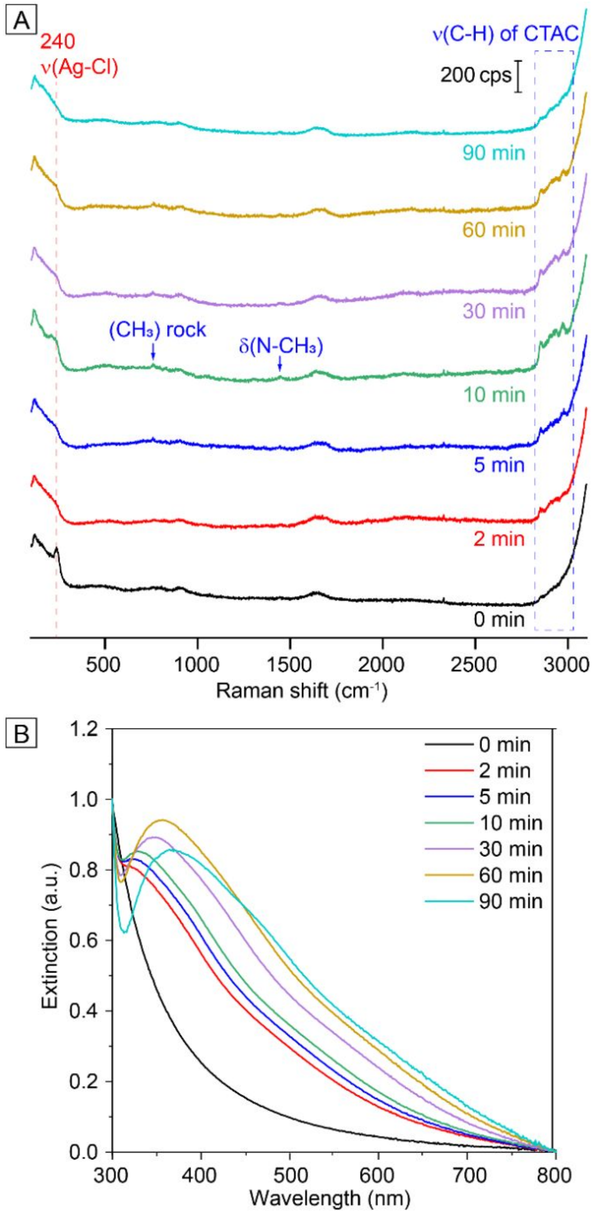

Figure S5. (A) Raman/SERS spectra and (B) UV-vis spectra showing the reduction of AgCl by H_2Asc in aqueous CTAC in the absence of preformed Ag seeds at different time points (t = 0−90 min). As shown in the A, before adding H_2Asc (t = 0 min), we could only resolve the Raman peak of AgCl at 240 cm^{-1}. After adding H_2Asc, the SERS signal from CTA^+ gradually became visible and reached its maximum strength after 10 min and then gradually decreased. This trend can be ascribed to the SERS enhancement brought about by the Ag nanocrystallites generated on the surface of AgCl nanoparticles in the absence of preformed Ag seeds, as well as the hot spots formed between them. The transfer of electrons from H_2Asc to the Ag nanocrystallites also caused chemical enhancement. As the Ag nanocrystallites started to grow, both H_2Asc and the AgCl solid

would be consumed, reducing the number of hot spots. At the same time, the peak associated with AgCl at 240 cm^{-1} disappeared at t = 2 min after adding H_2Asc. This change could be ascribed to the formation of a large number of Ag nanocrystallites on the surface of AgCl, as supported by the increase in optical extinction near 532 nm in the UV-vis spectrum. The Ag nanocrystallites obstructed the passing of the excitation light while attenuating the Raman signals from AgCl. The UV-vis spectra in the B also indicated the gradual consumption of AgCl over time. Although the Ag nanocrystallites grew with time, they remained very small at t = 90 min, as the position of their major LSPR peak was below 400 nm. Compared to the SERS spectra of seed-mediated growth, the SERS signals from CTA^+ and AgCl in the A, as well as their magnitudes of enhancement, were significantly weaker even after the growth had been initiated. The results demonstrated that the significant enhancement in SERS signals after adding H_2Asc mainly came from the preformed Ag seeds with a size of about 35 nm. The Ag nanocrystallites formed *in situ* on the AgCl nanoparticles in this control experiment were too small to provide SERS activity compatible to that of the preformed cubic seeds added into the growth mixture.

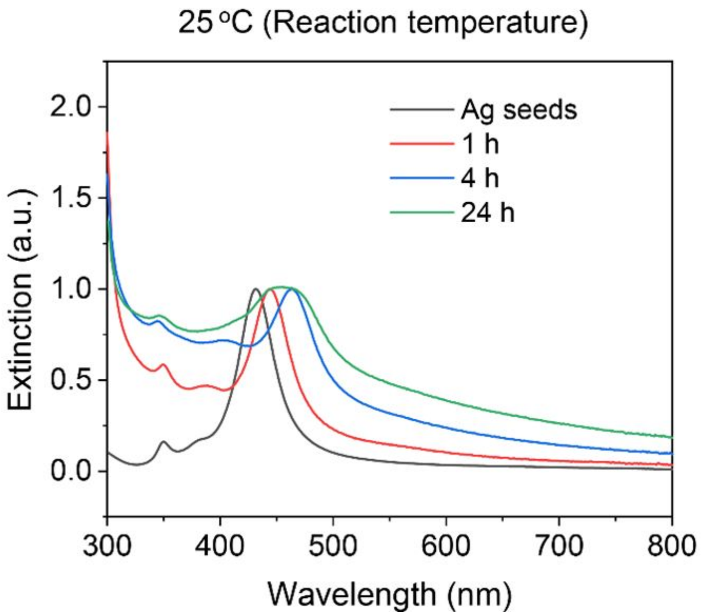

Figure S6. UV–vis spectra taken from aqueous suspensions of the Ag nanocubes obtained at different stages (t = 0−24 h) of a standard synthesis conducted in the presence of 100 μL of the seed suepension at 0 and 25 °C, respectively.

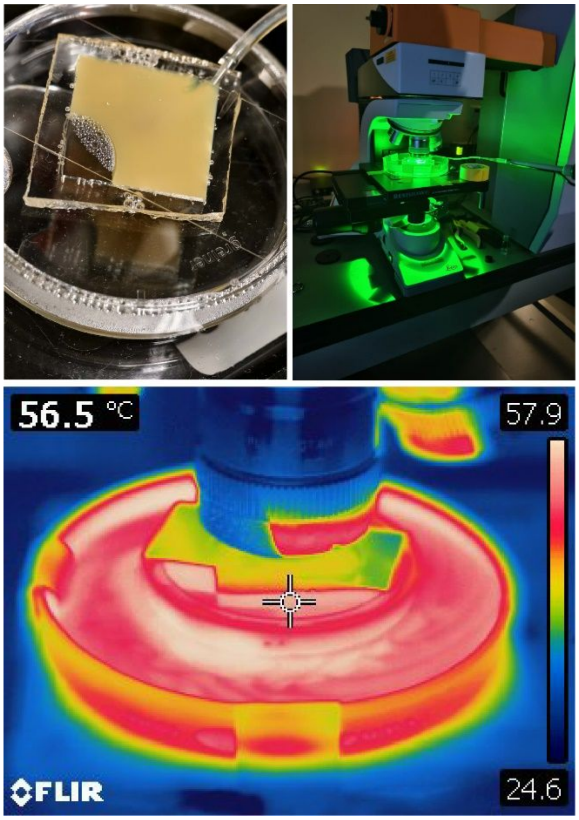

Figure S7. Digital photographs of the setup used for *in situ* SERS measurement and infrared image of the PDMS cell containing the reaction mixture during the *in situ* SERS measurement.
